# Special Issue—“Bio-Based Materials: Contribution to Advancing Circular Economy”

**DOI:** 10.3390/polym14183887

**Published:** 2022-09-17

**Authors:** Maya Jacob John, Sabu Thomas

**Affiliations:** 1 Centre for Nanostructures and Advanced Materials, Council for Scientific and Industrial Research (CSIR), Pretoria 0001, South Africa; 2 Department of Chemistry, Nelson Mandela University, Port Elizabeth 6001, South Africa; 3 International and Interuniversity Centre for Nano Science and Nano Technology, Mahatma Gandhi University, Kottayam 686560, India; 4 Basic Department of Biotechnology, School of Fundamental Biology and Biotechnology, Siberian Federal University, 79 Svobodnyi Av., 660041 Krasnoyarsk, Russia

Bio-based materials have a significant role to play in the implementation of a functional circular economy. The circular economy stresses the reduced use of raw materials, the reuse of products, waste streams (where waste is converted to a valuable resource), and the recycling of products, the combined effect of which will ensure that materials are retained in the loop as opposed to a linear economy which is based on a manufacture, use, and disposal model. In a circular society, bio-based materials are reused, repaired, recycled, and remanufactured. A circular approach for plastics will address the issue of plastic waste pollution on land and in oceans and the adverse health effects that microplastics have on marine and human life. This can be achieved by methods to improve recycling and developing bio-based materials as an alternative to petroleum-based feedstock. The concepts of the bio-based economy and the circular economy are similar in that they reduce the demand for fossil carbon and enhance the use of waste and side streams. Hence, it is quite clear that bio-based materials (bioplastics and biocomposites) can make a critical contribution to the implementation of the circular economy.

In this Special Issue, leading researchers from academia and industry were invited to submit reviews or their latest research on topics aligned to the development of sustainable materials from renewable resources. Sustainable materials include waste-derived, recyclable, and biodegradable materials. Studies dealing with recycling, waste conversion to bio-based products, the development of bio-based composites, and surface treatments on cellulose fibres have been included in this issue. The manuscripts were subjected to a rigorous review process, after which there was a compilation of thirteen research articles, reflecting the latest trends in bio-based and compostable materials. This issue consists of three review articles and ten research articles. The review articles include an account on the plastic circular economy and applications of chitosan-based materials.

As guest editors of this Special Issue, we acknowledge all the authors and reviewers who have contributed to its publication. We would also like to thank the technical support team at MDPI for their assistance in preparing this Special Issue.

